# Acute Kidney Injury in Adult Patients With Hepatocellular Carcinoma After TACE or Hepatectomy Treatment

**DOI:** 10.3389/fonc.2022.627895

**Published:** 2022-05-24

**Authors:** Zhixiang Mou, Tianjun Guan, Lan Chen

**Affiliations:** Department of Nephrology, Zhongshan Hospital of Xiamen University, School of Medicine, Xiamen University, Xiamen, China

**Keywords:** AKI, risk, mortality, hepatocellular carcinoma, HCC

## Abstract

**Background:**

Acute kidney injury (AKI) is one of the most common complications in patients with cancer, yet the specific reasons, mechanisms, and the influence of AKI are not clear in hepatocellular carcinoma (HCC) after treatment. This meta-analysis aimed to find out the risk factors and the impact on mortality of AKI in adult patients with HCC after treatment using available published data.

**Methods:**

We performed a systemic literature search using PubMed, Web of Science, and Embase, encompassing publications up until November 30, 2021 (inclusive), with 17 cohort studies involving 11,865 patients that fulfilled the prespecified criteria for inclusion in the meta-analysis. The number of AKI/non-AKI patients identified by risk factors, the number of AKI/non-AKI-related deaths, the incidence rates, the mortality rates, and the irreversible rates of AKI were derived and analyzed using STATA.

**Results:**

Age, diabetes mellitus (DM), and the number of transarterial chemoembolization (TACE) sessions are risk factors for AKI in patients with HCC after TACE. On the other hand, male gender, age, DM, major resection of the liver, and operation-related transfusion are risk factors for AKI in patients with HCC after hepatectomy. The risk of mortality in those with renal failure due to AKI was up to 4.74 times higher than in those without AKI in a short-term observation period after TACE treatment.

**Conclusions:**

Attention should be paid to the risk of AKI in HCC patients with DM. The occurrence of AKI during TACE treatment is especially dangerous and should be considered a strong red flag, obviously with regard to the extremely high risk of death in a short period. Furthermore, studies are needed to detect more associations of AKI in patients with HCC.

## 1 Introduction

As a global health problem, hepatocellular carcinoma (HCC) is the sixth most common cancer and the second leading cause of cancer-related death in men and the sixth in women (1). Although liver transplantation (LTx) is the most effective among all the therapeutic options, only about 5% of HCC patients are eligible for this therapy due to the strict indications (2). According to the National Comprehensive Cancer Network (NCCN) Guidelines, partial hepatic resection is the preferred option in patients without severe liver cirrhosis (excluding patients with Child–Pugh scores in classes B and C); meanwhile, locoregional therapy is the preferred option in patients unsuitable for surgery, which includes ablation, arterially directed therapies, and external beam radiation therapy (EBRT) (3).

Acute kidney injury (AKI) is one of the most common complications in cancer patients (4, 5). It refers to a rapid (hours to days) deterioration of renal function, which results in the failure to excrete waste and to maintain fluid balance, which can be severe as to require renal replacement therapy (RRT) (6). The management of these patients is a significant therapeutic challenge for physicians, and the chance of receiving optimal treatment might be less for those with poor kidney function since mortality from AKI remains high, particularly in critically ill patients (6). Efforts made to prevent AKI progression may contribute to survival and reduce the possibility of progressing to chronic kidney disease (CKD). As a clinical syndrome that results from severe or persistent events that may act as triggers, any diagnostic approach to investigating AKI should take into account the associated epidemiology (6). Although a large cohort study based on a Danish population reported that the risk of developing AKI in 1 year was about 33% in patients with liver cancer (7), there is still a lack of research focused on the association between AKI and HCC after treatment, especially regarding locoregional therapy or hepatectomy—the two major treatment options for HCC patients. The few previous studies that described AKI had limitations of a small study size, collection of data from a single medical center, or discussion of the incidence rates of AKI in newly diagnosed cancer, which present obvious restrictions regarding the generalizability of the findings. More comprehensive analyses are urgently needed to examine their authentic relationship in order to help provide proper management and to improve the clinical outcomes.

This meta-analysis was conducted based on HCC patients receiving locoregional therapy and hepatectomy, aiming to examine the risk factors and the impact on mortality of AKI in these HCC patients using available published data.

## 2 Materials and Methods

### Search Strategy

The protocol for this meta-analysis has been registered in the International Prospective Register of Systematic Reviews (PROSPERO no. CRD42020183617). A systematic literature review was performed by two authors (MZX and LC) independently through PubMed, Web of Science, and Embase, employing the search terms “acute kidney injury” OR “acute renal failure” AND “hepatocellular carcinoma” OR “liver cancer” OR “hepatoma” and including publications up until November 30, 2021 (inclusive). The search terms “contrast induced nephropathy” (CIN) AND “hepatocellular carcinoma” were also used as the previous recognition of renal dysfunction in HCC to investigate the incidence of AKI among adult patients with HCC. Each study was evaluated for inclusion or exclusion in this analysis (see below). No language or date restrictions were applied. This meta-analysis was conducted and reported according to the guidelines of the Preferred Reporting Items for Systematic Reviews and Meta-Analyses (PRISMA; http://www.prisma-statement.org/).

### Study Selection Criteria: Risk Factors, Outcomes, and Follow-Up

Firstly, potential eligible studies must meet the Population, Interventions, Comparison and Outcomes (PICO) criteria to fulfill the purpose of this analysis. The inclusion criteria were as follows: HCC patients underwent locoregional therapy or liver resection; the original cohort studies provided data on the AKI events based on adult patients (age, ≥18 years) with HCC; the Child–Pugh score is in Child–Pugh class A or B; the clinical characteristics/prognosis related to AKI could be clearly identified by the number of patients; and the definitions of AKI or RRT were not considered.

The following studies were excluded: those regarding LTx for HCC; comprising patients who had end-stage renal disease or were undergoing RRT; AKI cannot be identified by the number of patients; including other types of hepatobiliary cancers; and case/case series reports including ≤10 patients. Research works from the same hospital were carefully evaluated for exclusion. No restrictions on language or year were applied in the full text.

### Data Extraction and Study Quality

To extract the necessary data from each included study, a spreadsheet template (Excel, Microsoft Corporation, Redmond, WA, USA) was established. After a careful review of each included article, the following data were collected: first author, publication year, regions, risk factors of AKI, the number of HCC, AKI, or irreversible renal failure (RF) patients, the number of AKI/non-AKI-related deaths, AKI definitions, and the observation period. Studies that did not base AKI on the number patients required careful calculation to maintain the accuracy. Some original data unpublished online were obtained from authors after communication (8, 9).

Quality assessment for the included studies was conducted using the Newcastle–Ottawa Quality Assessment Scale (NOS), which comprises three aspects (selection, comparability, and outcomes) and eight items (10). This scale enables the researchers to score studies from 0 to 9, whereby those with a score ≥6 were considered of high methodological quality.

### Definitions of AKI, Risk Factors, Locoregional Therapy, and Observation Period

Although they have been validated in numerous patients and seem to work similarly (6), there are still over 30 AKI definitions used in the literature (11). RIFLE (Risk of renal failure, Injury to the kidney, Failure of kidney function, Loss of kidney function, and End-stage kidney disease), AKIN (Acute Kidney Injury Network), and KDIGO (Kidney Disease Improving Global Outcomes) are the three widely accepted criteria for the definition of AKI (12, 13). In this meta-analysis, AKI was accepted in the case of the original study having identified its occurrence regardless of any definition.

According to the NCCN Guidelines, locoregional therapy comprises the following: 1) ablation, including radiofrequency, cryoablation, percutaneous alcohol injection, and microwave ablation; 2) arterially directed therapies, including bland transarterial embolization (TAE), transarterial chemoembolization (TACE), TACE with drug-eluting beads (DEB-TACE), and radioembolization (RE) with yttrium-90 (Y-90) microspheres (3).

All potential risk factors that possibly affect the renal function of patients with HCC after treatment should be identified and screened.

Long-term refers to prognosis being observed after 1 year from AKI, whereas short-term indicates observation being conducted within 3 months from AKI.

### Statistical Analysis

STATA statistical software (version 16.0; StataCorp LLC, College Station, TX, USA) was utilized for statistical analysis. In the analysis, random effects models and the DerSimonian–Laird method were applied to analyze dichotomous variables (the number of AKI/non-AKI patients identified by risk factors and the number of AKI/non-AKI-related deaths), continuous variables, and proportion variables (the incidence rates, mortality rates, and the irreversible rates of RF). Double arcsine transformation was applied for the meta-analysis of low proportion variables to ensure normality. The *I^2^* test was used to assess heterogeneity. Pooled risk ratios (RRs), the weighted mean difference (WMD), and their corresponding 95% confidence intervals (CIs) were used to evaluate the risk factors of developing or the risk of mortality with AKI. The *Z*-test was used to assess the significance of the pooled RRs/WMDs, and a forest plot was drawn to graphically display the results of all statistical analyses. Statistically significant heterogeneity among studies is defined as *χ^2^*-value <0.05 or *I^2^* test >50%. Subgroup analyses were performed to investigate the original source of significant heterogeneity, and a *Z*-test *p*-value <0.05 was considered a statistically significant difference.

## 3 Results

### Literature Search and Study Characteristics

**Figure 1:**
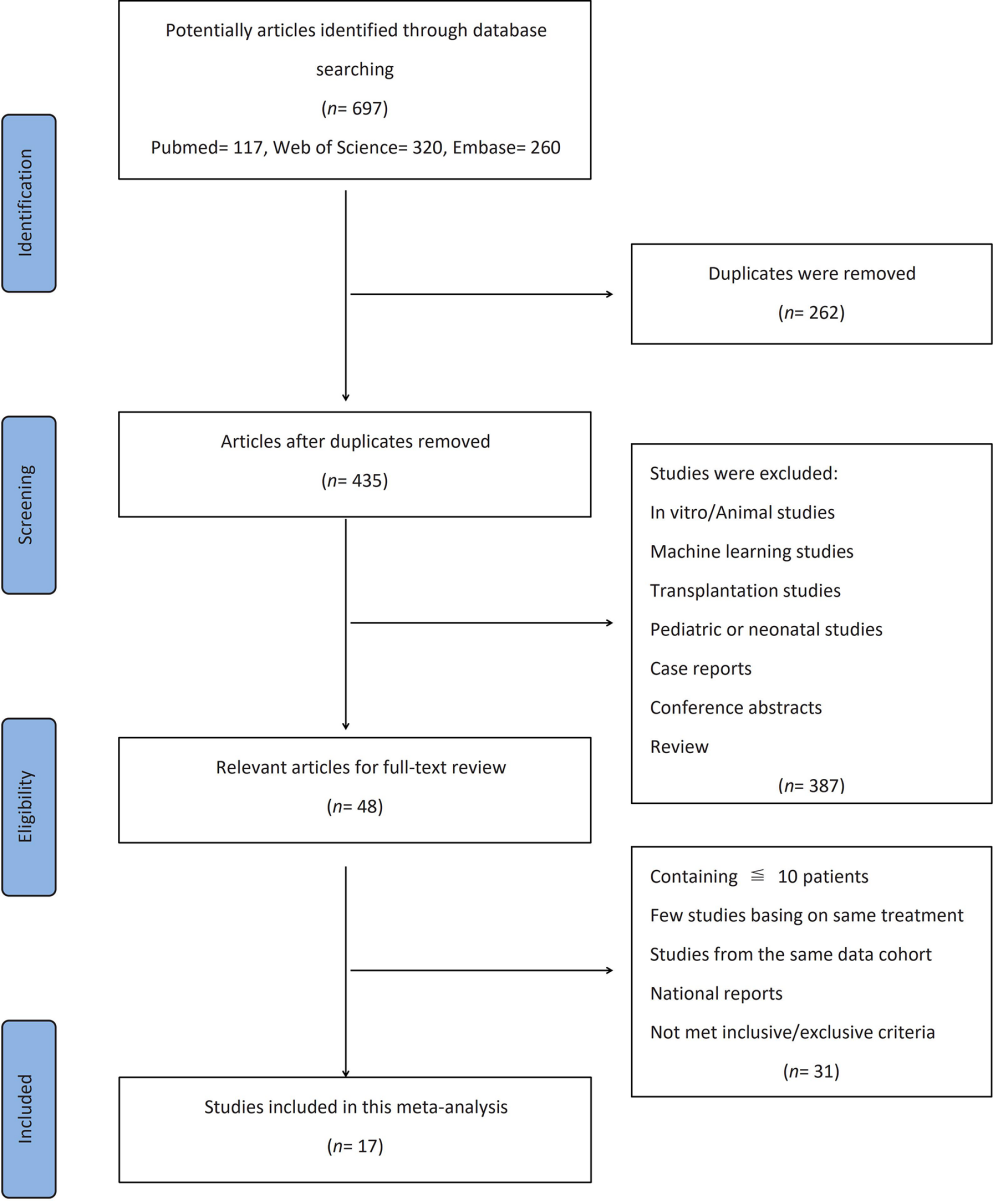
Flow diagram for this meta-analysis.

The flow diagram showing the selection process and the reasons for the exclusion of a systematic review is presented in detail in **Figure 1**. Three databases provided a total of records (PubMed, *n* = 117; Web of Science, *n* = 320; Embase, *n* = 260). After the exclusion of duplicates (262 records), the titles and abstracts of 435 articles were manually screened for eligibility. Then, studies on *in vitro*/animal, machine learning, transplantation, or pediatric/neonatal populations; case reports; conference abstracts; and review articles were excluded. Thereafter, the full texts of the 48 remaining articles were reviewed for eligibility. The remaining studies that included only ≤10 patients, not enough papers using the same treatment method, studies from the same cohort, and national reports (duplicated representative population) were also excluded after careful review.

Finally, 17 cohort studies involving a total of 11,865 patients that fulfilled the prespecified criteria were included in the meta-analysis (**Tables 1**, **2**). Among them, 10 studies were based on TACE, 1 study was based on TACE and TAE (these 11 studies would be analyzed together, hereinafter as “TACE”), and 6 studies were based on liver resection. Nine studies (52.9%) with a score ≥6 were considered of high quality according to the NOS criteria (**Table 3**). Sixteen studies (94.1%) reported the outcomes with clearly defined AKI, 14 studies (82.4%) reported at least one risk factor for developing AKI, while 10 studies (58.8%) reported AKI-related death.

**Table 1:** Characteristics of the studies about transarterial chemoembolization (TACE).

Study	Year	Region	Risk factors for AKI	No. of HCC patients	No. of AKI patients	Death with RF	AKI definitions	Observation period	Irreversible RF (inclusive of death with RF)
Huo et al. (14)	2004	Taiwan	Gender, age, multiple tumor, DM, HBsAg, TACE sessions	140	12	1	KDIGO	11 weeks	4
Huo et al. (15)	2004	Taiwan	Gender, age, multiple tumor, DM, HBsAg, TACE sessions, amount of contrast	235	56	25	KDIGO	Long term	27
Park et al. (16)	2008	Korea	Gender, age, multiple tumor, DM, HBsAg, TACE sessions, amount of contrast, NSAID	236	24	Short-term^a^: 1 Long-term^b^: 19	AKIN	Short-term Long-term	Short-term 6 Long-term 4
Hsu et al. (17)	2009	Taiwan	Gender, multiple tumor, DM, HBsAg	87	11	Short-term: 2 Long-term: 9	KDIGO	Short-term Long-term	Short-term: 4
Cho et al. (11)	2011	South Korea	N/A	91	18	5	Scr >25% within 2–4 days	In-hospital	N/A
Hayakawa et al. (9)	2014	Japan	N/A	115	8	1	Scr >25% within 2–3 days	N/A	N/A
Lee et al. (18)	2017	Taiwan	NSAID	1,132	72	N/A	N/A	N/A	N/A
Zhou et al. (19)	2018	China	Gender, age, DM, amount of contrast, NSAID	818	38	3	KDIGO	1 month	4
Lin et al. (8)	2019	Taiwan	Gender, multiple tumor, DM, HBsAg, TACE sessions	96	17	1	KDIGO	1 month	N/A
Sohn et al. (20)	2020	South Korea	N/A	347	37	N/A	ICA-AKI	Short-term	N/A
Si et al. (21)	2021	China	Gender	284	28	N/A	Scr >25% within 2–3 days	4 days	N/A

HCC, hepatocellular carcinoma; AKI, acute kidney injury; RF, renal failure; DM, diabetes mellitus; TACE, transarterial chemoembolization; NSAID, non-steroidal anti-inflammatory drug; N/A, not applicable; AKIN, Acute Kidney Injury Network; KDIGO, Kidney Disease Improving Global Outcomes; ICA, International Club of Ascites.

a Short-term: the results were observed within 3 months.

b Long-term: the results were observed after 1 year.

**Table 2:** Characteristics of the studies about hepatectomy.

Study	Year	Region	Risk factors for AKI	No. of HCC patients	No. of AKI patients	Death with RF	AKI definitions	Observation period	Irreversible RF (inclusive of death with RF)
Tsai et al. (22)	2014	Taiwan	DM, major resection	5,924	62	N/A	ICD-9-CM 584	N/A	N/A
Lim et al. (23)	2016	France	Gender, age, DM, cirrhosis, major resection, transfusion	457	67	Short-term^a^: 25 Long-term^b^: 46	KDIGO	Short-term Long-term	32
Ishikawa et al. (24)	2017	Japan	Gender, age, DM, cirrhosis, major resection, transfusion	228	27	N/A	AKIN	3 years	N/A
Moon et al. (25)	2017	Korea	Gender, DM, transfusion	1,173	77	N/A	AKIN	1 year	42
Bressan et al. (26)	2018	Canada	Gender, DM, cirrhosis, major resection	80	16	2	AKIN	1 month	N/A
Xu et al. (27)	2018	China	Gender, age, DM, cirrhosis, major resection	422	48	N/A	KDIGO	3 months	N/A

HCC, hepatocellular carcinoma; AKI, acute kidney injury; RF, renal failure; DM, diabetes mellitus; N/A, not applicable; HRS, hepatorenal syndrome; KDIGO, Kidney Disease Improving Global Outcomes; AKIN, Acute Kidney Injury Network.

a Short-term: the results were observed within 3 months.

b Long-term: the results were observed after 1 year.

**Table 3:** Newcastle–Ottawa Scale for assessing the quality of cohort studies.

Study	Representativeness of the exposed cohort	Selection Selection of the non-exposed cohort	Ascertainment of exposure	Demonstration of the outcome of interest being not present at the start of study	Comparability Comparability of cohorts on the basis of the design or analysis	Outcomes Assessment of outcome	Was follow-up long enough for outcomes to occur?	Adequacy of follow-up of cohorts	Score
									
Huo et al., 2004	★	★	★		★	★	★		6
Huo et al., 2004	★	★	★		★	★	★		6
Park et al., 2008	★	★	★		★	★	★		6
Hsu et al., 2009	★	★	★		★		★		5
Cho et al., (2011)	★	★	★	★	★				6
Hayakawa et al., (2014)	★	★	★	★	★				7
Tsai et al., (2014)	★	★	★		★				4
Lim et al., (2016)	★	★	★	★	★	★	★		7
Lee et al., 2017	★	★	★		★				4
Moon et al., 2017	★	★	★	★	★				5
Ishikawa et al., 2017	★	★	★	★	★	★			6
Zhou et al., 2018	★	★	★		★	★			5
Bressan et al., 2018	★	★	★	★	★		★		6
Xu et al., 2018	★	★	★	★	★				5
Lin et al., 2019	★	★	★	★	★	★			6
Sohn et al., 2020	★	★	★			★	★		5
Si et al., 2021	★	★	★				★		4

The Newcastle-Ottawa Scale quality instrument is scored by awarding a point for each answer that is marked with a star below. Total points are 4 points for Selection, 2 points for Comparability, and 3 points for Outcomes.

### Patient Characteristics

In this study, 11,865 adult patients with HCC between July 1996 and December 2019 were identified as being eligible for analysis. Of these patients, 30.2% (*n* = 3,581) received TACE treatment and 69.8% (*n* = 8,284) received hepatectomy.

For the analysis of HCC-AKI patients receiving TACE, eight studies reported on 2,377 men and 651 women suffering from HCC, four studies recorded the ages of 1,429 patients with HCC, five studies reported on 373 HCC patients with multiple tumor, six studies reported diabetes mellitus (DM) as a comorbidity for 216 patients with HCC, five studies reported on 517 HCC patients with HBsAg(+), three studies reported on 1,362 patients receiving non-steroidal anti-inflammatory drug (NSAID) treatment, four studies provided the number of TACE sessions on 707 patients, and three studies described the amount of contrast on 1,289 patients. In this series analysis, patients with a history of renal insufficiency were excluded.

For the analysis of HCC-AKI after hepatectomy, 6,197 (74.8%) men and 2,087 (25.2%) women with HCC were recorded in six studies (three studies recorded the ages of 1,107 patients), with 3,236 of them undergoing DM simultaneously. Five studies reported on 1,564 patients who underwent major resection of the liver and 5,547 patients who had minor resection, while 226 patients who needed transfusion due to surgery were recorded in three studies. In this series analysis, patients with a history of end-stage renal disease were excluded.

### Analysis of the Risk Factors for AKI in HCC Patients Receiving TACE Treatment

**Figure 2:**
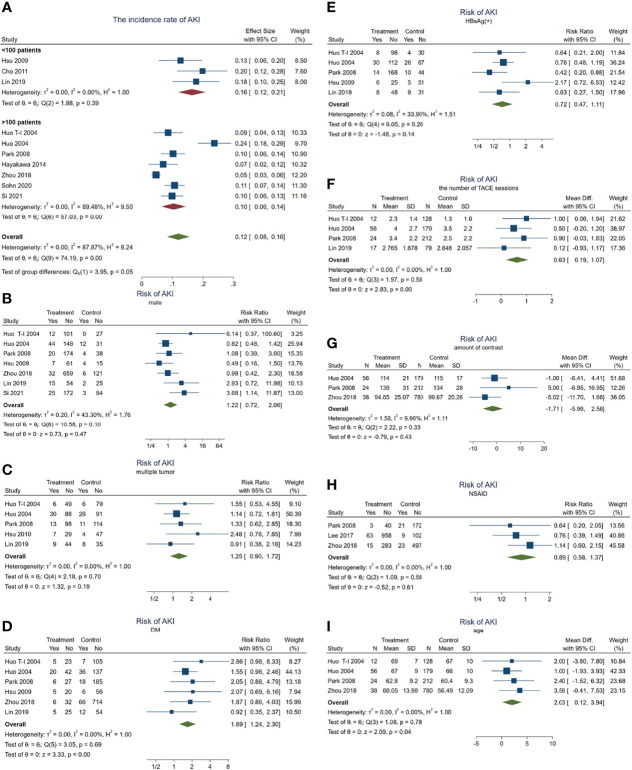
Forest plots of the included studies assessing the risk factors for AKI in patients with HCC who received TACE treatment. The *solid vertical line* indicates no effect. The *horizontal lines* represent the 95% confidence intervals (CIs). **(A)** Incidence rates of AKI in these patients. **(B–I)** Supposing male gender **(B)**, multiple tumor **(C)**, DM **(D)**, HBsAg(+) **(E)**, the number of TACE sessions **(F)**, amount of contrast **(G)**, NSAID use **(H)**, and age **(I)** as risk factors. AKI, acute kidney injury; HCC, hepatocellular carcinoma; TACE, transarterial chemoembolization; DM, diabetes mellitus; NSAID, nonsteroidal anti-inflammatory drug.

Overall, 249 patients with HCC developed AKI during TACE treatment. The incidence rate of AKI in these HCC patients was about 11.9% (95% CI = 8.3–15.5, *p* < 0.001, *I^2^* = 87.9%, *χ^2^p* < 0.001) (see **Figure 2A**). Subgroup analyses were performed according to the number of enrolled HCC patients to examine the original source of significant heterogeneity. We found that there was no significant heterogeneity observed in the <100 patient subgroup (*I^2^* = 0, *χ^2^p* = 0.39); however, the significant heterogeneity was still high in the >100 patient subgroup (*I*^2^ = 89.5%, *χ*^2^*p* < 0.001), which indicated that the significant heterogeneity may have come from the sample size.

Subsequently, we carried out a series meta-analysis to detect the risk factors for AKI. When male gender was taken as a risk factor for AKI, the results showed no significant difference (pooled RR = 1.22, 95% CI = 0.72–2.06, *p* = 0.47) (**Figure 2B**) and significant heterogeneity (*I*^2^ = 43.3%, *χ*^2^*p* = 0.1), indicating that male gender is not a risk factor for developing AKI in patients with HCC receiving TACE.

Subsequent analysis indicated that multiple tumors (inclusive of diffuse tumor; pooled RR = 1.25, 95% CI = 0.90–1.72, *p* = 0.187, *I*^2^ = 0, *χ*^2^*p* = 0.701) (**Figure 2C**), positive HBsAg (pooled RR = 0.72, 95% CI = 0.47–1.11, *p* = 0.14, *I*^2^ = 34%, *χ*^2^*p* = 0.195) (**Figure 2E**), the amount of contrast (WMD = −1.71, 95% CI = −5.99 to 2.56, *p* = 0.43, *I*^2^ = 9.96%, *χ*^2^*p* = 0.33) (**Figure 2G**), and NSAID use (pooled RR = 0.89, 95% CI = 0.58–1.37, *p* = 0.606, *I*^2^ = 0, *χ*^2^*p* = 0.58) (**Figure 2H**) were also not risk factors for AKI. Significant heterogeneity was not observed.

On the other hand, when the meta-analysis was conducted taking DM as a risk factor, the results showed that the risk of AKI in patients with DM was 1.69 times higher than in those without DM (pooled RR = 1.69, 95% CI = 1.24–2.3, *p* = 0.001) (**Figure 2D**), but no significant heterogeneity was observed (*I*^2^ = 0, *χ*^2^*p* = 0.692).

In addition, having more TACE sessions (pooled WMD = 0.63, 95% CI = 0.20–1.06, *p* = 0.004, *I*^2^ = 0, *χ*^2^*p* = 0.499) (**Figure 2F**) or older age (pooled WMD = 2.03, 95% CI = 0.12–3.94 years, *p* = 0.04, *I*^2^ = 0, *χ*^2^*p* = 0) (**Figure 2I**) would contribute to developing AKI more easily. These results demonstrated that age, DM, and the number of TACE sessions may act as risk factors for AKI.

### 3.3 Dangers of AKI in HCC Patients Receiving TACE Treatment

**Figure 3:**
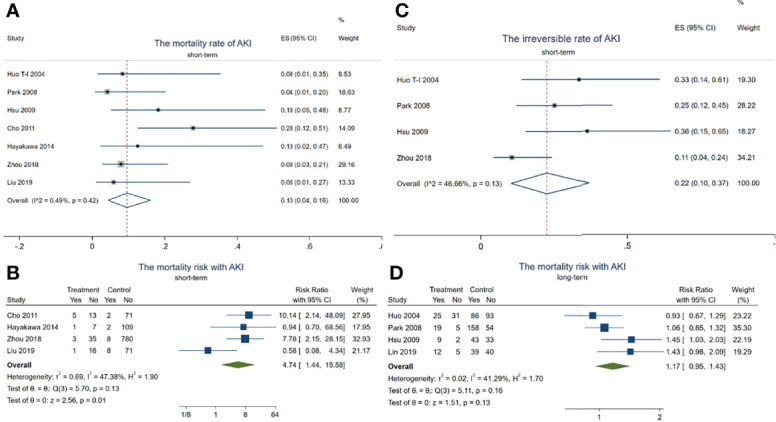
Forest plots of the included studies assessing the risk of acute kidney injury (AKI) in patients with hepatocellular carcinoma (HCC) who received transarterial chemoembolization (TACE) treatment. The *solid vertical line* indicates no effect. The *horizontal lines* represent the 95% confidence intervals (CIs). **(A)** Mortality rates of AKI in these patients. **(B)** Mortality risk with AKI during the short term. **(C)** Irreversible rates of AKI in these patients. **(D)** Mortality risk with AKI after long-term observation.

In total, 14 died out of 128 HCC patients with AKI in the short-term observation (within 3 months). The mortality rate of AKI in HCC patients receiving TACE was about 10.0% (95% CI = 4–16, *p* < 0.001, *I*^2^ = 0.49%, *χ*^2^*p* = 0.42) (see **Figure 3A**) during this period.

Although there was no difference in the mortality risk with AKI and without AKI (pooled RR = 1.17, 95% CI = 0.95–1.43, *p* = 0.13, *I*^2^ = 41.29%, *χ*^2^*p* = 0.16) (**Figure 3D**) after long-term observation (after 1 year), the mortality risk with AKI reached up to 4.74 times higher than in those without AKI in the short-term period (pooled RR = 4.74, 95% CI = 1.44–15.58, *p* = 0.01, *I*^2^ = 47.38%, *χ*^2^*p* = 0.13) (**Figure 3B**). In addition, 18 HCC patients with AKI progressed to irreversible kidney injury during the short-term period, with the irreversible rate of AKI being about 22% (95% CI = 4–16, *p* < 0.001, *I*^2^ = 0.49%, *χ*^2^*p* = 0.42) (see **Figure 3C**). These results indicated that TACE-related AKI is not only a dangerous signal related to death but also presents a high possibility of progressing to CKD in these patients within a short period.

### Analysis of the Risk Factors for AKI in HCC Patients After Hepatectomy

**Figure 4:**
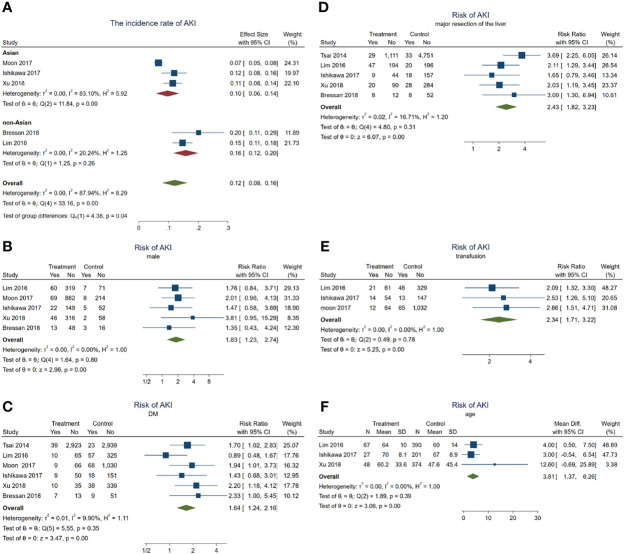
Forest plots of the included studies assessing the risk factors for acute kidney injury (AKI) in patients with hepatocellular carcinoma (HCC) after hepatectomy. The *solid vertical line* indicates no effect. The *horizontal lines* represent the 95% confidence intervals (CIs). **(A)** Incidence rates of AKI in these patients. **(B–F)** Supposing male gender **(B)**, diabetes mellitus (DM) **(C)**, major resection of the liver **(D)**, receiving transfusion **(E)**, and age **(F)** as risk factors.

In total, 235 patients with HCC progressed to AKI after hepatectomy. The incidence rate of AKI in these HCC patients was about 12% (95% CI = 8–16, *p* = 0.04, *I*^2^ = 87.94%, *χ*^2^*p* < 0.001) (see **Figure 4A**). Subgroup analyses were performed according to the different ethnicities to examine the original source of the significant heterogeneity. We observed no significant heterogeneity in the non-Asian subgroup (*I*^2^ = 20.24%, *χ*^2^*p* = 0.26); however, the significant heterogeneity was still high in the Asian subgroup (*I*^2^ = 83.1%, *χ*^2^*p* < 0.001), indicating that the significant heterogeneity may have come from the ethnicity difference.

Subsequently, a series meta-analysis was also carried out to examine the risk factors for AKI in these patients. Men presented 1.83 times higher risk than women (pooled RR = 1.83, 95% CI = 1.23–2.74, *p* < 0.001, *I*^2^ = 0, *χ*^2^*p* = 0.8) (**Figure 4B**), having DM was 1.64 times higher than that without DM (pooled RR = 1.64, 95% CI = 1.24–2.16, *p* < 0.001, *I*^2^ = 9.9%, *χ*^2^*p* = 0.35) (**Figure 4C**), after major resection of the liver showed 2.43 times higher risk than after minor resection (pooled RR = 2.43, 95% CI = 1.82–3.23, *p* < 0.001, *I*^2^ = 16.71%, *χ*^2^*p* = 0.31) (**Figure 4D**), and having received transfusion during hepatectomy had 2.34 times higher risk than without a need for transfusion (pooled RR = 2.34, 95% CI = 1.71–3.22, *p* < 0.001, *I*^2^ = 0, *χ*^2^*p* = 0.78) (**Figure 4E**). Finally, older patients would also develop AKI (pooled WMD = 3.81, 95% CI = 1.37–6.26, *p* = 0, *I*^2^ = 0, *χ*^2^*p* = 0.39) (**Figure 2F**) more frequently.

In a word, male gender, age, DM, major resection, and transfusion may act as risk factors for AKI during hepatectomy for HCC.

Due to the limited data available, the risk of AKI in patients with HCC after hepatectomy was not analyzed.

## 4 Discussion

The major findings of these published data based on the meta-analyses were as follows: firstly, DM is a risk factor for AKI in patients with HCC either receiving TACE or hepatectomy, which means that close attention should be paid to HCC patients with DM for risk of AKI during these treatments. Secondly, the number of TACE sessions is another risk factor for AKI in patients with HCC receiving TACE treatment. Moreover, male gender, major resection of the liver, and transfusion due to hepatectomy are other risk factors for AKI in HCC patients after hepatectomy. Lastly, the incidence of AKI during TACE treatment is especially dangerous: the risk of mortality with AKI was up to 4.74 times higher than in those without AKI in the short-term period.

A lot of advanced HCC patients require locoregional treatment due to inadequate hepatic reserve, liver-confined disease, being inoperable by performance status, comorbidity, or having uncertain extrahepatic diseases (3). The efficiency and safety of the TACE procedure have been improved for several decades; it is also considered the main treatment option for patients who had four or more HCCs and with liver function assessed as Child–Pugh class A or B (28). Previously recognized as CIN, TACE treatment of patients with HCC is the third leading cause of hospital-acquired AKI, which contributes to prolonged hospital stay and readmission rates (11). The specific reasons, mechanisms, and the influence of AKI in these patients are still unclear. HCC often develops from chronic liver disease that has already progressed to advanced cirrhosis, which may contribute to the development process of AKI. Abnormal systemic hemodynamics, splanchnic arterial vasodilatation, and extrahepatic vasoconstriction are possibly involved in cirrhosis-related AKI (29). The application of iodinated radiocontrast agents potentially is an acute event further exaggerating the already disturbed hemodynamics and/or renal vasoconstriction in advanced cirrhosis, finally leading to renal dysfunction. In addition, nephrotoxic drugs such as iodinated radiocontrast agents, adriamycin, and lipiodol can lead to renal microcirculatory dysfunction, cell apoptosis, or endothelial injury independently (6). On the other hand, renal endothelial cells would be impaired even at quite early exposure to a hyperglycemic milieu, whereas prolonged hyperglycemia would promote the mesenchymal transition and fibrosis of endothelial cells (30), resulting not only in endothelial dysfunction and aggravating kidney fibrosis but also in being vulnerable to nephrotoxicity by radiocontrast agents. Once DM is a comorbidity, the nephrotoxicity of radiocontrast agents will obviously be strengthened due to either transient or persistent hyperglycemic conditions. These theories were further demonstrated in our research, where the incidence of AKI during TACE treatment is not radiocontrast agent dose-dependent, and a small dose is strong enough to cause AKI in HCC-DM patients. The age-related susceptibility of AKI in older individuals has been reported both in TACE and hepatectomy, and the reason may be that older age enhances renal vulnerability as well (9, 26).

The hemodynamic alterations following liver resection are similar to advanced cirrhosis (31). In addition, AKI in patients with HCC ensues from hepatectomy probably more related to transient/prolong renal hypoperfusion or ischemia, while major resection of the liver or preoperative/postoperative transfusion could significantly aggravate this course due to the persistence of extensive blood loss and reduction of oxygen delivery. Firstly, ischemia would induce a significant functional impairment or structural damage of small renal tubular and vascular malfunction (30), which serves as the initiation of systemic inflammatory response activation and leads to renal inflammation injury and microcirculation dysfunction (32). In addition, microvascular damage could obviously affect endothelial cell expansion, apoptosis, or necrosis, in turn leading to microvascular obstruction, further inhibiting post-ischemic reperfusion and delaying kidney regeneration. Furthermore, ischemia would diminish the total surface intrarenal vascular area, along with endothelial–mesenchymal transition, together leading to the loss of important intrinsic physiological defense mechanisms and finally increasing the vulnerability of nephrons to oxygen-free radicals (30, 32). Therefore, even a minor or a laparoscopic liver resection should not be considered a less harmless operation and the prevention of intraoperative hemorrhage should also be paid the same attention, and vice versa.

Different from the hypothesis of ischemia–reperfusion injury, investigation of the association between diabetes-induced endothelial dysfunction and ischemia leading to the vulnerability of the kidney is rare. However, this relationship has been found based on several animal research works: a diabetic mouse model showed a higher vulnerability to ischemia than did non-diabetic controls, and ischemia was even induced quite early (33). On the other hand, non-diabetic rats completely recovered from functional impairment and tissue damage caused by renal ischemia, while diabetic rats failed within about 2 months observation (34). Tumor protein 53 (*TP53*) is the most frequently mutated tumor-suppressor gene in HCC. Inactivating mutations of *TP53* possibly present in 20% of HCCs in western countries, while they present in >50% of HCCs in aflatoxin B1 (AFB1)-exposed regions (35–38). Peng and colleagues demonstrated that P53 played a protective role against AKI in diabetic animal models, either in diabetic mice inducing P53-specific siRNAs or in proximal tubule-specific P53-knockout mice inducing diabetes (33). This may be one explanation for the different incidence rates of AKI consistently observed between the different ethnicities in this study.

Despite ischemia–reperfusion injury or the hemodynamic instability of renal perfusion, transfusion of red blood cells may be an independent risk factor for postoperative AKI: impaired oxygen unloading of hemoglobin due to 2,3­diphosphoglycerate deficiency, less deformability of stored red blood cells leading to the obstruction of smaller capillaries, increase in circulating free iron from stored red blood cell hemolysis, release of procoagulant phospholipids, and the accumulation of pro-inflammatory phospholipids together exaggerate the existing inflammatory response and lead to sepsis-associated AKI (39–41). Hepatorenal syndrome (HRS) describes a reversible AKI in patients with advanced hepatic failure, including advanced cirrhosis. Its varied performance depends on the volume and quality of the remnant liver after hepatectomy (steatosis/cirrhosis). Hepatic microcirculation is already impaired by steatosis or cirrhosis, and the liver presents more mitochondrial dysfunction and is less resistant to ischemia–reperfusion injury. TACE or hepatectomy intervention is a probable acute incident prompting the sudden decrease in the glomerular filtration rate (GFR) and renal perfusion. The potential pathophysiological mechanisms comprise significant splanchnic vasodilation and elevated abdominal pressure accompanied by ascites, causing overactivity of the renin–angiotensin–aldosterone system (RAAS) and sympathetic nervous system (SNS), followed by vasoconstriction/structural damage of the kidney and intravascular hypovolemia, accompanied by necrosis/apoptosis of tubular cells, which would drop off and obstruct the lumen, together causing complete deterioration of the GFR (31–41).

The study has several limitations. Firstly, analyses of the influence and the potential for publication bias could not be effectively performed due to the limited number of original studies (<10) for every meta-analysis. Secondly, statistical heterogeneity was always observed in the meta-analysis. One potential origin of the heterogeneity may be the ethnicity. When a subgroup analysis was performed according to the different ethnicities, the results showed no heterogeneity in the incidence rates of AKI in the non-Asian subgroup, but the statistical heterogeneity existing in the Asian group needs further exploration. As previously mentioned, P53 plays important roles both in HCC and AKI during DM; future studies could probably focus on HCC-AKI in diverse ethnicities. The accurate moment of earlier diagnosis of AKI by any definition is indeed difficult to establish in these patients due to the varied efficacy–efficiency balance of biomarker measurements, which is one of the reasons the International Club of Ascites (ICA) spent several years developing the new expert consensus on the diagnosis and treatment of AKI in patients with liver cirrhosis. This contention may be another source of the heterogeneity. Finally, since the clinical data are from publications and have limitations in terms of availability, not only could further sub-analyses not be performed (TNM stage, duration of hepatectomy, and tumor size, among others), but AKI in patients with HCC receiving other treatments (radiofrequency, microwave ablation, or systemic therapy) could also not be analyzed. Incidentally, further studies are still needed to support the conclusions and demonstrate more associations of AKI in HCC patients.

In conclusion, age, DM, and the number of TACE sessions are risk factors for AKI in patients with HCC receiving TACE, while age, male gender, DM, major resection of the liver, and operation-related transfusion are risk factors for AKI in patients with HCC after hepatectomy. Finally, the occurrence of AKI during TACE treatment is especially dangerous and should be considered a strong red flag, obviously with regard to the extremely high risk of death in a short period. Furthermore, studies are needed to detect more associations of AKI in patients with HCC (especially in patients receiving other treatments).

## Data Availability Statement

The original contributions presented in the study are included in the article/supplementary material. Further inquiries can be directed to the corresponding author.

## Author Contributions

CL had the idea for the study and formulated its design, having had full access to all data in the study, and takes responsibility for the integrity of the data and the accuracy of the data analysis. ZM and TG contributed to data acquisition and the writing of the report. ZM contributed to critical revisions of the report and to the statistical analysis. All authors commented on previous versions of the manuscript. All authors contributed to the article and approved the submitted version.

## Funding

This research was supported by the Xiamen Municipal Bureau of Science and Technology (grant no. 3502Z20199173).

## Conflict of Interest

The authors declare that the research was conducted in the absence of any commercial or financial relationships that could be construed as a potential conflict of interest.

## Publisher’s Note

All claims expressed in this article are solely those of the authors and do not necessarily represent those of their affiliated organizations, or those of the publisher, the editors and the reviewers. Any product that may be evaluated in this article, or claim that may be made by its manufacturer, is not guaranteed or endorsed by the publisher.
